# PIM1 regulates glycolysis and promotes tumor progression in hepatocellular carcinoma

**DOI:** 10.18632/oncotarget.3534

**Published:** 2015-03-12

**Authors:** Carmen Oi-ning Leung, Carmen Chak-lui Wong, Dorothy Ngo-yin Fan, Alan Ka-lun Kai, Edmund Kwok-kwan Tung, Iris Ming-jing Xu, Irene Oi-lin Ng, Regina Cheuk-lam Lo

**Affiliations:** ^1^ Department of Pathology, The University of Hong Kong, Hong Kong, China; ^2^ State Key Laboratory for Liver Research, The University of Hong Kong, Hong Kong, China

**Keywords:** PIM1, HCC, metastasis

## Abstract

Hepatocellular carcinoma (HCC) is characteristically one of the most rapidly proliferating tumors which outgrows functional blood supply and results in regional oxygen deprivation. Overexpression of PIM1, a serine/threonine kinase, has been identified recently in human cancers. Knowledge on PIM1 in HCC is however, scarce. By immunohistochemical analysis on 56 human primary HCC samples, we observed overexpression of PIM1 in 39% of the cases. In two independent cohorts of paired primary and extra-hepatic metastatic HCC tissues, PIM1 expression was higher (p=0.002) in the extra-hepatic metastatic HCC tissues as compared with the corresponding primary HCCs. PIM1 was markedly up-regulated in multiple HCC cell lines in hypoxic condition (1% O_2_) versus normoxia (20% O_2_). Silencing of PIM1 suppressed HCC cell invasion *in vitro* as compared to non-target control, and decreased HCC cell proliferation *in vitro* and tumor growth and metastatic potential *in vivo*. Knockdown of PIM1 significantly reduced glucose uptake by HCC cells and was associated with decreased levels of p-AKT and key molecules in the glycolytic pathway. Taken together, PIM1 is up-regulated by hypoxia in HCC and promotes tumor growth and metastasis through facilitating cancer cell glycolysis. Targeting PIM1 may have potential role in the management of HCC.

## INTRODUCTION

Hepatocellular carcinoma (HCC) is one of the most malignant cancers worldwide and highly prevalent in China and Southeast Asia. Characteristically, HCC is rapidly proliferating and often outpaces its functional blood supply, leading to regional oxygen deprivation and resulting in frequent intratumoral hypoxia. Furthermore, HCC palliative therapies (transarterial [chemo]embolization [TACE/TAE]) that involve restriction of blood flow to the tumor bulk induce severe hypoxia. The major mechanism by which HCC cells adapt to hypoxia is through induction of the transcription factor, hypoxia inducible factor (HIF) [1]. O_2_ acts as a substrate for the prolyl hydroxylation of HIF-1/2α [2], which subsequently enables Von-Hippel-Lindau (VHL) to mediate ubiquitin-proteasomal degradation [3]. Therefore, inhibition of propyl hydroxylase (PHD) by dimethyloxalylglycine (DMOG) could stabilize HIF-1/2α expression under normoxic condition.

In human cancers, the hypoxic tumor microenvironment has been widely studied and is recognized as an important condition driving cancer metastasis [4]. In HCC, HIF-1α and HIF-2α were consistently found to be over-expressed in tumor tissues [5, 6]. Moreover, expression of *vascular endothelial growth factor* (VEGF), a well-characterized HIF target, is associated with poor prognosis in HCC patients [5].

PIM1 belongs to a group of constitutively activated serine/threonine kinases firstly identified as the *P*roviral *i*ntegrating site for *M*oloney murine leukemia virus, which is a cause of murine leukemia [7, 8]. The three members in the PIM family, PIM1 (chromosome 6), PIM2 (chromosome X) and PIM3 (chromosome 22), physiologically mediate cytokine signaling of hematopoietic cells [9]. In neoplastic conditions, PIM1 and PIM2 are involved in the oncogenesis of acute myeloid leukemia [10]. Knowledge on PIM1 in solid cancers has been emerging in recent years. PIM1 was found to be overexpressed in gastric [11] and prostate cancers [12], which was associated with aggressive clinicopathological features.

Notably, hypoxia is one of the mechanisms regulating PIM1 expression. PIM1 was induced by hypoxia in pancreatic cancer cell lines, in which PIM1 is stabilized by hypoxia by preventing it from ubiquitin-mediated proteasomal degradation [13]. Apart from hypoxia, microRNAs such as miR33a were found to regulate PIM1 expression at post-transcriptional level [14]. Heat shock proteins (HSP) alter PIM1 expression by post-translational mechanisms. Binding to HSP70 induces ubiquitylation and proteasomal degradation of PIM1, while heat shock protein 90 (HSP90) protects PIM1 from proteasomal degradation [15, 16].

Although PIM1 is the founding member of the PIM family, the role of PIM1 in HCC remains elusive. In this study, we aimed to address the key questions on the expression pattern of PIM1 in HCC, the regulatory mechanism of PIM1 expression with special reference to hypoxia, and the functional characterization of PIM1 in HCC. We found that PIM1 was overexpressed in primary HCCs and the expression level was higher in the extra-hepatic HCC metastatic tissues than the primary tumors. Silencing of PIM1 inhibited HCC proliferation and invasion *in vitro* and tumor growth and metastasis *in vivo*. PIM1 expression was up-regulated under hypoxic conditions mainly by inhibition of proteasomal degradation in a HIF-1α independent manner. Furthermore, PIM1 reprogramed HCC metabolism and facilitated tumor cell glycolysis, which in turn sustained tumor growth and enhanced tumor invasion.

## RESULTS

### PIM1 is overexpressed in primary and metastatic HCC tissues

**Figure 1:**
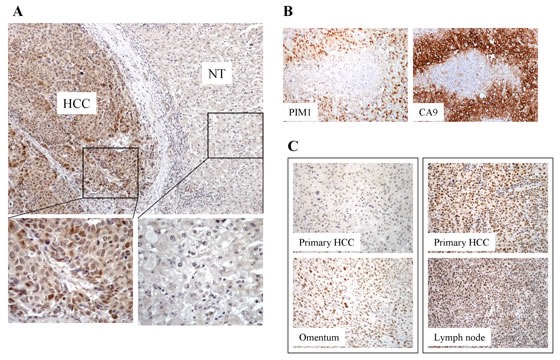
(A) Expression of PIM1 in HCC tissues by immunohistochemistry. PIM1 was overexpressed in HCC. Positive nuclear and cytoplasmic staining was observed in the HCC tumors. No staining was present in the corresponding non-tumorous (NT) tissues. (B) Immunohistochemical staining of PIM1 and CA9 in HCC tissues. (C) PIM1 immunohistochemical expression in paired primary and extra-hepatic metastatic HCC tissues. *Left panel* - PIM1 staining was negative in the primary HCC tissue but was positive in the corresponding metastatic HCC tissue in the omentum; *Right panel* - PIM1 staining was positive in both primary HCC and the corresponding metastatic HCC tissue in the lymph node.

By immunohistochemical analysis (whole-section) in 56 human HCC samples, 22 (39%) cases showed PIM1 overexpression in primary HCC tissues as compared to the corresponding non-tumorous (NT) liver tissues (Figure 1A). Overexpression is defined by cytoplasmic and/or nuclear staining in HCC tumor cells but not in the adjacent non-tumoral liver cells. In the clinical samples, moderate to strong nuclear and cytoplasmic staining was observed in the HCC tissues; while all NT tissues did not show specific staining. The clinicopathological parameters and the staining pattern were detailed in Supplementary Table S1. We also tested the immunoexpression of cirrhotic liver tissues not associated with tumors, and none showed positive staining in the hepatocytes (Supplementary Figure 1). With additional immunohistochemical staining for Carbonic Anhydrase 9 (CA9) and Glucose Transporter 1 (GLUT-1), both being well known HIF targets, in the primary HCC samples, we observed expression of CA9 and/or GLUT-1 in HCC tissues in majority (20/22) of the PIM1-positive cases(Figure 1B). To further determine the clinical significance of PIM1 expression in HCC tissues, we examined PIM1 immunoexpression in a separate independent set of 19 paired primary and extra-hepatic HCC tissues (whole-section) to explore the potential role of PIM1 in HCC metastasis (Table 1). Among these 19 cases, PIM1 expression was found in 11 (57.9%) primary HCC tissues and 18 (95%) metastatic HCC tissues, respectively (Figure 1C). Notably, in 7 (37%) of the 19 cases, PIM1 was negative in the primary HCCs but positive in the corresponding metastatic tissues. We further extended the immunohistochemical analysis on 24 pairs of primary and metastatic tissues in a tissue microarray. Four (17%) and 12 (50%) cases were found to express PIM1 in the primary and metastatic tissues respectively. The expression rate was lower than that from the whole section cohort most probably as a result of sampling error in a tissue microarray setting. However similarly, a significant proportion of cases (9/24; 37.5%) showed negative PIM1 immunostaining in the primary tissues but positive staining in the metastases. PIM1 expression is significantly higher in metastatic HCC tissues than in the primary combining results from the two cohorts (p=0.002), which suggests that PIM1 may be up-regulated in the metastatic process of HCC. A similar significant result was still obtained after eliminating some cases in the two cohorts with a longer time interval between the resection of primary and metastatic tumors. The clinicopathological parameters and the staining pattern of the two paired primary and metastatic cohorts were detailed in Supplementary Table S2.

**Table 1:** Clinical features of the 19 cases with paired primary and metastatic HCC samples

Case number	Sex/age	Primary liver disease	Site of extra-hepatic metastasis
1	M/55	CHB	Lungs
2	M/61	CHB	Lungs
3	M/58	CHB	Peritoneum
4	F/83	CHC	Brain
5	M/51	CHB	Thoracic lymph node
6	M/36	CHB	Lungs
7	M/55	CHB	Brain
8	M/79	CHB	Lungs
9	M/54	CHB	Omentum
10	F/52	CHB	Lungs
11	F/65	CHB	Portal lymph node
12	M/66	CHB	Peritoneal lymph node
13	M/53	CHB	Lungs
14	M/69	CHB	Paracaval lymph node
15	F/54	CHB	Brain
16	M/50	CHB	Lungs
17	M/53	CHB	Lungs
18	M/78	CHB	Adrenal gland
19	M/51	CHB	Portal vein thrombus

CHB: chronic hepatitis C; CHC: chronic hepatitis C

### Silencing of PIM1 inhibits tumor growth and metastasis *in vivo*

**Figure 2:**
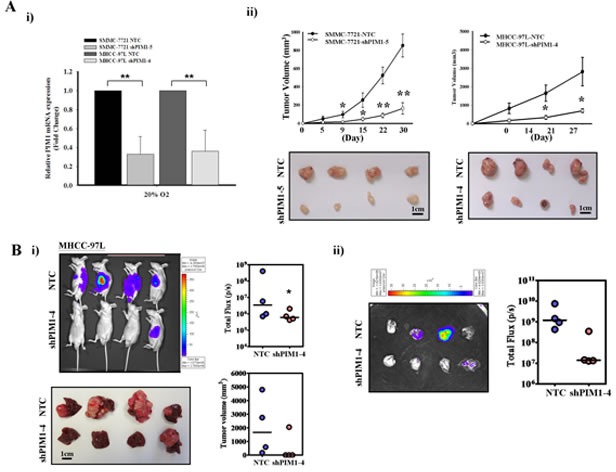
Silencing of PIM1 suppressed the tumor volume and metastatic ability *in vivo* (A) i) Quantitative PCR analysis of PIM1 expression in NTC and PIM1 stable knockdown clones in SMMC-7721 (shPIM1-5) and MHCC-97L (shPIM1-4), respectively. ii) Subcutaneously inoculation of NTC and shPIM1-5 (SMMC-7721) or shPIM1-4 (MHCC-97L). Tumor growth volume upon inoculation measured with calipers; tumors were excised from the two groups of mice. (B) i) Xenogen imaging of nude mice orthotopically implanted with tumors derived from MHCC-97L-NTC and shPIM1-4 at 6^th^ week upon implantation, with tumor incidence rate indicated. Livers from the two groups of mice at 6^th^ week upon implantation, with tumor volume indicated respectively. ii) Ex-vivo imaging of lung from the two groups of mice, with pulmonary metastasis rate indicated.

In light of the expression pattern of PIM1 by immunohistochemical analysis, we next investigated the tumorigenicity of PIM1 *in vivo* by comparing the tumor growth using PIM1 knockdown approach. Based on the endogenous expression of PIM1 in HCC cells lines, we established PIM1 stable knockdown clones in SMMC-7721 and MHCC-97L cell lines (Figure 2Ai & Supplementary Figure 2). The knockdown effect was specific for PIM1 but not for PIM2 and PIM3 as demonstrated by qPCR (Supplementary Figure 3). Using subcutaneous injection of tumor cells in nude mice, knockdown of PIM1 in both HCC cell lines significantly reduced the tumor growth *in vivo* (Figure 2Aii) in SMMC-7721 and MHCC-97L cell lines, respectively, as compared with their NTC (p=0.003 and 0.036 for SMMC-7721 and MHCC-97L, respectively). To examine the effect of the metastatic enhancing ability of PIM1 on HCC cells in a more physiological environment, orthotopic liver implantation model was employed using MHCC-97L cell line, a metastatic HCC cell line. The tumor-forming incidence was lower in PIM1 stable knockdown clone (1/4), as compared with NTC (4/4). The corresponding incidence of lung metastasis was also lower in PIM1 stable knockdown clone (Figure 2B).

### Hypoxia induces expression and nuclear translocation of PIM1 protein in HCC cells

**Figure 3:**
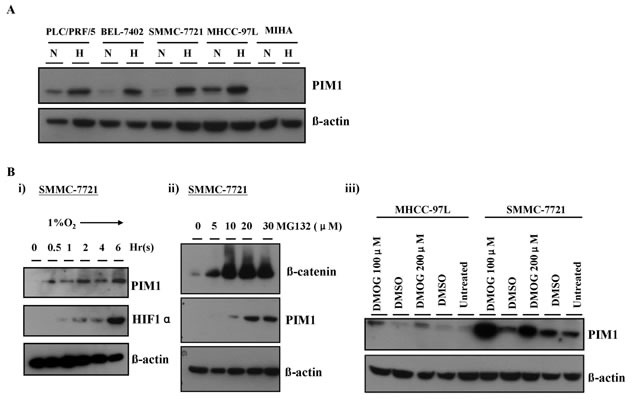
PIM1 expression was up-regulated by hypoxia, which was HIF-1α independent (A) PIM1 expression in HCC cell lines and MIHA. N: normoxia (20% O_2_) H: hypoxia (1% O_2_). (B) i) PIM1 protein expression was up-regulated at around 30 minutes and preceded that of HIF-1α in SMMC-7721. ii) Treatment of SMMC-7721 with proteasome inhibitor MG132 in normoxia stabilized PIM1 protein. iii) Treatment with propyl hydroxylase inhibitor DMOG in SMMC-7721 and MHCC-97L also stabilized PIM1 protein.

Since hypoxia can drive cancer metastasis [4], the expression of PIM1 in HCC cell lines in normoxic (20% O_2_) and hypoxic (1% O_2_) conditions were analyzed. In normoxia, PIM1 was expressed at variable levels in PLC/PRF/5, BEL-7402, SMMC-7721 and MHCC-97L cell lines upon Western blot analysis. When the HCC cells were exposed to the hypoxic condition, the PIM1 expression was markedly up-regulated in all these cell lines, while the immortalized normal liver cell line MIHA did not express PIM1 whether under normoxia or hypoxia (Figure 3A).

With immunohistochemistry on the clinical HCC samples, we observed both nuclear and cytoplasmic staining in the HCC cells. Besides, nuclear translocation of PIM1 in hypoxia was reported in pancreatic cell lines [17]. Therefore, we performed cell fractionation and Western blotting to examine the subcellular localization of PIM1 protein in normoxia and hypoxia. We observed that in normoxia, PIM1 was located in both the cytoplasm and nuclei of HCC cells, whereas hypoxia markedly enhanced nuclear translocation of PIM1 protein (Supplementary Figure 4).

### Up-regulation of PIM1 in hypoxia is HIF-1α-independent and partly mediated by proteasomal degradation pathway

To interrogate whether hypoxia induction of PIM1 occurred at transcriptional, post-transcriptional, translational or post-translational level, we performed a time-point experiment to examine the PIM1 protein expression in HCC cells exposed to hypoxia and compared with that of HIF-1α. Up-regulation of PIM1 protein expression occurred at ~30 minutes of hypoxic treatment in SMMC-7721 cells and preceded that of HIF-1α. PIM1 expression gradually increased and leveled off (Figure 3Bi). The findings suggest that PIM1 may not be a direct target of HIF-1α.

To investigate whether PIM1 protein was subjected to ubiquitin-proteasomal degradation, which a common post-translational regulatory mechanism of protein expression, we treated the HCC cell lines (SMMC-7721 and MHCC-97L) with a proteasomal inhibitor MG-132 in normoxia. We found that PIM1 protein level markedly increased and was stabilized upon treatment with MG-132 (Figure 3Bii), suggesting that PIM1 is degraded by ubiquitin-proteasomal pathway in normoxia. In attempt to further dissect this proteasomal degradation mechanism, we examined whether PIM1 protein was stabilized upon treatment of prolyl hydroxylase (PHD) inhibitor, dimethyloxaloylglycine (DMOG). We found that inhibiting PHD activity by DMOG also stabilized PIM1 protein expression in normoxic condition in SMMC-7721 and MHCC-97L cells (Figure 3Biii), suggesting that PHD may mediate the degradation of PIM1 in normoxia.

### Suppression of PIM1 reduces HCC cell proliferation and invasion

**Figure 4:**
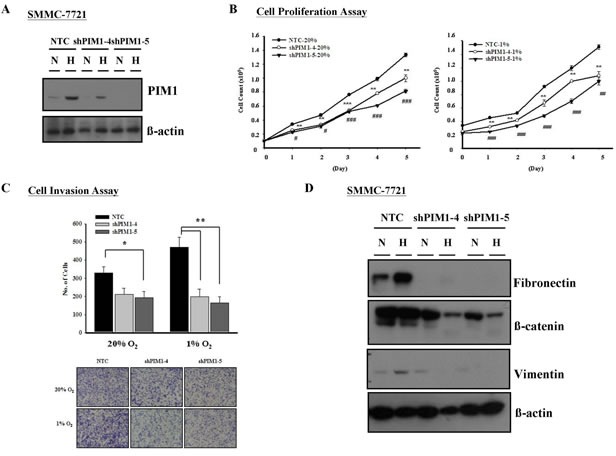
Silencing of PIM1 inhibited cell proliferation and invasiveness in HCC (SMMC-7721) (A) Western blot analysis of PIM1 expression in PIM1 stable knockdown and NTC clones. (B) Silencing of PIM1 suppressed cell proliferation in both normoxic and hypoxia conditions (* NTC vs shPIM1-4; # NTC vs shPIM1-5; */# p<0.05; **/## p<0.01; ***/### p<0.001). (C) *Upper panel* - Knockdown of PIM1 inhibited cell invasiveness in normoxic and hypoxic conditions (20% O_2_: NTC vs shPIM1-4 p=0.053; NTC vs shPIM1-5 p=0.030; 1% O_2_: NTC vs shPIM1-4 p=0.008; NTC vs shPIM1-5 p=0.003). *Lower panel* - Representative pictures of transwell after staining with 1% crystal violet. (D) Expression of epithelial-mesenchymal markers in PIM1 stable knockdown clones shPIM1-4 and shPIM1-5 of SMMC-7721.

Further to the results from *in vivo* experiments and immunohistochemical analysis on human HCC samples, we proceeded to consolidate the roles of PIM1 in HCC *in vitro* using the PIM1 stable knockdown clones in SMMC-7721 cells (Figure 4A). We performed functional assays to investigate the effect of modulated PIM1 expression on HCC tumor growth and progression under normoxia and hypoxia. Silencing PIM1 significantly suppressed the proliferation of HCC cells in both normoxic and hypoxic conditions with cell proliferation assay (Figure 4B). With Matrigel invasion assay, silencing of PIM1 in SMMC-7721 cells reduced the cell invasive ability under both normoxic and hypoxic conditions, with more pronounced effects in the latter (p <0.010 in both knockdown clones versus p = 0.053 and 0.030 in normoxia) (Figure 4C). The results suggest that PIM1 enhances HCC cell invasion, and more significantly in hypoxic condition. Since we observed that PIM1 was associated with increased invasive ability of HCC cells, we attempted to explore the correlation between PIM1 expression and epithelial-mesenchymal transition (EMT) phenotype of HCC cells. By Western blotting, we found that knockdown of PIM1 was associated with decreased protein expression levels of fibronectin, β-catenin and vimentin under hypoxia, while the effect in normoxia was variable among the 3 markers (Figure 4D). These suggest that PIM1 may play an important role in activating EMT under hypoxic condition.

### Knockdown of PIM1 dampens glycolysis of HCC cells

**Figure 5:**
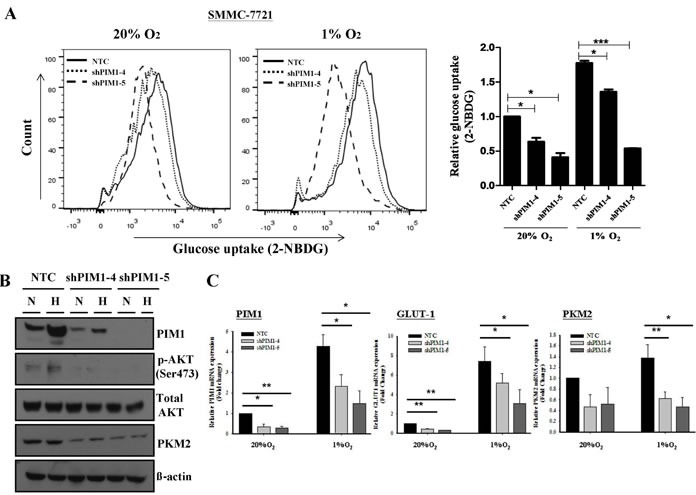

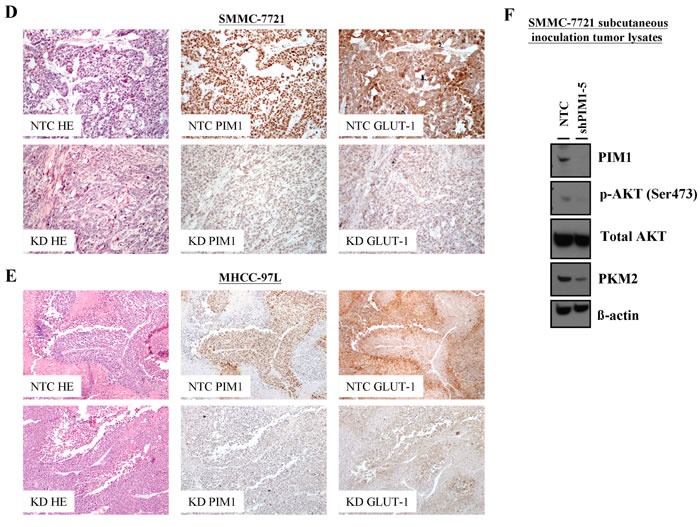
Knockdown of PIM1 suppressed the glycolytic metabolism in SMMC-7721, possibly through the regulation of p-AKT (A) Glucose uptake experiment using 2-NBDG flow cytometry analyses (*left panel)* and relative glucose uptake (*right panel)*. (B) Expression of p-AKT, total AKT and pyruvate kinase M2 (PKM2) in SMMC-7721 stable PIM1 knockdown clones shPIM1-4 and shPIM1-5. N: normoxia (20% O_2_) H: hypoxia (1% O_2_). (C) Expression of GLUT-1 and PKM2 by qPCR in PIM1 stable knockdown clones of SMMC-7721. (D) Immunohistochemical staining of PIM1 and GLUT-1 of HCC tissues in NTC and PIM1 knockdown (KD) clones from subcutaneous inoculation (SMMC-7721) and (E) orthotopic implantation (MHCC-97L) of nude mice. (F) Expression of PIM1, p-AKT, total AKT and PKM2 of SMMC-7721 tumor lysates from NTC and shPIM1-5 after subcutaneous inoculation in nude mice.

We demonstrated that PIM1 was up-regulated by hypoxia in HCC and enhanced the cell proliferation and invasiveness of HCC cells especially in a hypoxic microenvironment. We then questioned if PIM1 was involved in the regulation of glycolysis in HCC cells. By glucose uptake assays, we observed an obvious decrease in glucose uptake in PIM1 knockdown clones compared to NTCs in normoxia. The decrease became even more drastic in hypoxia (Figure 5A). The results suggest that PIM1 is involved in sustaining both aerobic and anaerobic glycolysis of HCC cells.

### AKT is a downstream target of PIM1 in HCC glycolysis

Next, we wished to identify the potential downstream target of PIM1. Sustaining glycolysis in HCC cells may play a pivotal role since energy is required for cancer cells to proliferate and invade. Various studies have shown that the PI3K/AKT pathway is important in mediating glycolysis of cancer cells. Phosphorylation of AKT at ser473 position increases AKT activity [18] and phosphorylated AKT in turn activates key enzymes in the glycolytic pathway including the phosphofructokinase 2 (PKF2) [18] and hexokinase 2 (HK2) [19]. In addition, AKT up-regulates Glucose Transporter 1 (GLUT-1) expression in cancer cells [18]. In our experiment with Western blotting, knockdown of PIM1 resulted in decreased p-AKT and pyruvate kinase M2 (PKM2) levels (Figure 5B), while the total AKT level remained static. In addition, the transcript levels of both GLUT-1 and PKM2, key molecules in the glycolytic pathway, were down-regulated in PIM1-stable knockdown clones as compared with the NTCs (Figure 5C). In view of these findings, we proceeded to examine the expression of various markers in the glycolytic pathway in the tumor tissues from our *in vivo* mouse model. Coherent with our *in vitro* data, GLUT-1 immunoexpression in HCC cells was lower from the PIM1 knockdown clones in both subcutaneous inoculation (SMMC-7721) and orthotopic implantation (MHCC-97L) in mice when compared with NTC (Figures 5D & E). On Western blot analysis with the tumor lysates from subcutaneous inoculation in mice (SMMC-7721), there was a down-regulation of p-AKT (Ser473) and PKM2 expression in the PIM1 knockdown clone compared with NTC (Figure 5F). The results suggest that PIM1 regulates HCC glycolysis through AKT and its known downstream effectors.

## DISCUSSION

In this study, we demonstrated the overexpression of PIM1 protein in human HCCs in a cohort of clinical samples. Furthermore, PIM1 was more frequently expressed in extra-hepatic HCC metastases than the corresponding primary HCCs. By Western blotting, we observed up-regulation of PIM1 in hypoxia in multiple HCC cell lines, accompanied by enhanced nuclear translocation of the protein. Results from our functional assays established a significant role of PIM1 in HCC cell proliferation and invasion, particularly under hypoxic conditions. The role of PIM1 in HCC growth and metastasis was substantiated by *in vivo* experiments. We also provided evidence on the role of PIM1 in reprogramming HCC metabolism through glycolysis, and demonstrated that AKT is a potential downstream target of PIM1. On dissection of the regulatory mechanism of PIM1 regulation by hypoxia, we found that it was HIF-1α-independent and probably through inhibition of the PHD activity, hence inhibiting the proteasomal degradation of the PIM1 protein.

Cancer metabolism is an important link between tumor microenvironment and tumor progression [20]. Cancer cells seize survival benefits through molecular mechanisms mechanistically linked to metabolic reprogramming, which may include ATP generation, evasion of apoptosis, sustained angiogenesis, and avoidance of immunosurveillance [20]. Hypoxia promotes cancer metastasis partly by altering the metabolism of cancer cells including glycolysis [21]. Glycolysis is an important mechanism for cancer cells to generate energy and sustain proliferation and aggressive features. In hypoxia, glycolysis is increased. Cyclical hypoxia leads to constitutively active glycolysis even in the presence of adequate oxygen, when cancer cells adopt aerobic glycolysis (Warburg's effect) [20, 22, 23].

How hypoxia regulates cancer glycolysis has been a topic for investigation. In this regard, HIF-1 plays an important role in reprogramming cancer cells to glycolytic metabolism under hypoxia and in regulating the expression of GLUT-1 [24-26]. The co-operation between HIF-1 and c-myc in aerobic glycolysis was studied and elaborated [27]. p53 is also known to alter the expression of GLUT in cancer cells [28]. GLUT-1 is overexpressed in HCC tissues and facilitates hepatocarcinogenesis [29, 30]. Apart from HIF, the PI3K/AKT pathway is crucial in regulating the expression of glucose transporters and activates some key enzymes in the glycolytic process [18]. In our current study, the novel role of PIM1 in cancer glycolysis sheds light on how PIM1 sustains HCC growth and progression through manipulation of cell metabolism. Not being a HIF target, PIM1 enhances AKT-mediated glycolysis by activating the phosphorylation of AKT and hence its downstream targets to produce energy and maintain HCC growth and progression in the hypoxic tumor microenvironment.

From the results of our time point experiments and those with MG132 treatment, PIM1 protein up-regulation in hypoxia occurred at post-translational level as quickly as 30 minutes upon hypoxic treatment of HCC cells. From previous studies, it was suggested that tumor hypoxia can be acute or chronic and there has been evidence that acute hypoxia (cycle times ranging from 20-60 min) in tumors is more readily observed than chronic hypoxia [31-33]. In this connection, PIM1, as a quick responder to tumor hypoxia, may be capable of facilitating HCC cells to adapt to the harsh survival environment and promote tumor growth and progression.

Our study has dissected the role of PIM1 in HCC and enriched the understanding on the genetic changes in response to the hypoxic microenvironment in HCC. Apart from regulating HCC proliferation, invasion and glycolysis, other potential functional roles of PIM1 in HCC may be related to chemoresistance. Hypoxia is also known to induce chemoresistance in cancers [21]. In pancreatic cancer, PIM1 contributes to chemoresistance [13]; hence, it is worthwhile to further explore whether PIM1 carries a similar function in HCC, a deadly cancer that is notorious for its chemoresistance.

PIM1 is known to enhance cancer cell survival by phosphorylating a number of target genes such as p21^waf1^, p27Kip1 and Cdc25C [34-36]. The PI3K/AKT pathway is also known to be crucial in cancer cell survival [37]. The potential interaction between PIM1 and AKT regarding the regulation of HCC cell proliferation in normoxia and hypoxia could possibly be elucidated in future studies.

From our data, we observed nuclear translocation of PIM1 protein under hypoxia in HCC cells. Hence we postulate that the nuclear translocation of PIM1 protein under hypoxic conditions may help to protect the protein from being degraded in the cytoplasm. The nuclear fraction might serve as a reservoir of the protein for its function as a phosphorylating kinase. However, the mechanism for the nuclear-cytoplasmic shuffling remains to be elucidated.

The utility of specific PIM1 inhibitors or monoclonal antibodies to treat cancer cells has been documented in prostate cancer and leukemia [38-40]. Given the significant role of PIM1 in HCC in various aspects and since PIM1 is not a direct HIF-1α target, specific PIM1 inhibitors might play a unique function in intervening HCC metabolism and progression. The efficacy of such inhibitors, specifically targeting at tumor growth and metastasis as well as cell metabolism, is yet to be determined in HCC.

## MATERIALS AND METHODS

### Human HCC samples

Human HCCs and their corresponding non-tumorous liver samples in the cohort of 56 primary HCCs were obtained from patients with liver resection between 1992 and 2001 at Queen Mary Hospital, Hong Kong and randomly selected for the present study. All specimens collected were either snap-frozen in liquid nitrogen and stored at −80°C, or fixed in 10% formalin for paraffin embedding. Frozen sections from tumorous and non-tumorous liver samples were cut and stained for histological examination to ensure homogenous cell population of tissues. Use of human specimens was approved by the institutional review board of the University of Hong Kong/Hospital Authority Hong Kong West Cluster. Among the 56 patients, 45 were male and 11 female. The age ranged from 28-82 years. Forty-six (82.1%) patients were serum positive for hepatitis B surface antigen (HBsAg); 9 were negative.

### Cell lines models

Human HCC cell lines BEL-7402, SMMC-7721, PLC/PRF/5, MHCC-97L and immortalized normal liver cell line MIHA were used. BEL-7402, SMMC-7721 and MIHA were obtained from Shanghai Institute of Cell Biology (Shanghai, China). PLC/PRF/5 was purchased from American Type Culture Collection (Manassas, VA). MHCC-97L was a gift from Professor Z.Y. Tang at Fudan University (Shanghai, China). BEL-7402 and SMMC-7721 were maintained in Dulbecco's Modified Eagle Minimal Essential Medium (DMEM) with high glucose (GIBCO-BRL, Grand Island, NY). PLC/PRF/5 was maintained in MEM (GIBCO-BRL) with 1mM sodium pyruvate (Sigma Aldrich, St. Louis, MO), whereas MHCC-97L and MIHA were maintained in DMEM high-glucose with sodium pyruvate. All media were supplemented with 10% fetal bovine serum (FBS), penicillin at 100 units/mL and streptomycin at 100 μg/mL (Invitrogen, Gaithersburg, MO). All cell lines were cultured at 37°C in a humidified incubator with 5% carbon dioxide (CO_2_). For normoxic conditions, the cells were incubated in normal incubator (considered as 20% O_2_). For hypoxic experiments, the cells were cultured in hypoxic chamber supplemented with 1% O_2_.

### Immunohistochemistry

Immunohistochemical stain was performed on formalin-fixed, paraffin-embedded sections using the labeled horseradish peroxidase (HRP) method. Paraffin sections of 4 μm thick were mounted on poly-L-lysine-coated slides, deparaffinized and rehydrated in gradient alcohol series and distilled water. The slides were immersed in 10mM EDTA Tris-HCl antigen retrieval buffer (pH9.0) and boiled for 15 minutes, followed by slow cooling down at running tap water. Endogenous peroxidase activities were quenched by 3% H_2_O_2_ for 30 minutes, followed by rinsing twice in ddH_2_O and once with 0.1% Tween-20 in TBS. The sections were immersed in serum free-protein block solution (Dako, Glostrup, Denmark) for 15 minutes and incubated with monoclonal antibody against PIM1 (1:250, Abcam, Cambridge, UK), GLUT1 (1:500, Abcam), carbonic anhydrase 9 (CA9) (1:1000, Abcam), diluted in antibody diluent at 4°C overnight. Negative controls were incubated with antibody diluent only. The sections were then thoroughly washed with 0.1% Tween-20 in TBS five times, and incubated with Envision^TM^ HRP-conjugated secondary antibody (Dako) for 1 hour, followed by washing of five times with 0.1% Tween-20 TBS. Positive signals were visualized using 3, 3′-diaminobenzidine (Dako). Nuclei were counterstained with hematoxylin.

### Establishment of PIM1 stable knockdown HCC cells

Short-hairpin RNAs (shRNAs) targeting PIM1 were purchased from Sigma-Aldrich (Mission® shRNA bacterial glycerol stock). The clone IDs were TRCN0000010118 (clone 1), TRCN0000010117 (clone 2), TRCN0000010115 (clone 3), TRCN0000196869 (clone 4) and TRCN0000199011 (clone 5). In short, they were indicated as shPIM1-1 to shPIM1-5. To package the virus, 2×10^5^ 293FT cells (passage 10-12) were seeded in 6-well plate 24 hours prior to transfection. Either 1 μg of shPIM1 or non-target control (NTC) plasmid were co-transfected with 1.5 μg of packaging plasmid mix (System Biosciences, Mountain View, CA) using Lipofectamine 2000 (Invitrogen). The viral supernatant was collected 24-hour post-transfection and 400 l were used to infect SMMC-7721 and MHCC-97L in polybrene (Sigma-Aldrich) supplemented media at 5 μg/mL. After 48 hours of infection, the infected cells were selected with puromycin (Sigma-Aldrich) in gradient manner (5 μg/mL at 1^st^ week, then 2.5 μg/mL at 2^nd^ week and later maintained in 1 g/mL). The success of silencing of PIM1 in HCC cells was confirmed both normoxic and hypoxic conditions using western blotting.

### *In Vivo* subcutaneous xenograft inoculation

The study protocol was approved by the Committee of the Use of Live Animals in Teaching and Research at the University of Hong Kong. 1×10^6^ of either NTC or shPIM1 cells (SMMC-7721 and MHCC-97L) were resuspended 100 μl PBS and injected subcutaneously in both sides of posterior flanks (right flank: NTC; left flank: shPIM1) of 4- to 6-week old male athymic nude mice (BALB/CAnN-nu). Tumor sizes were measured every 3 days by calipers, and tumor volume (mm^3^) were calculated by the formula length × width^2^ × 0.5. The animals were sacrificed after 4 weeks and tumor bulks were harvested. All experiments were performed according to the Animals (Control of experiments) Ordinance (Hong Kong). The experiments were performed twice.

### Orthotopic liver implantation of HCC cells in nude mice

Orthotopic implantation was performed as previously described [41]. One million of either stable NTC or shPIM1 in firefly luciferase-labeled MHCC97L were resuspended in 15 μl matrigel and orthotopically injected into the left hepatic lobe of 6-week-old male nude mice. Tumor growth was monitored by bioluminescent approach through injecting 100 mg/kg of D-luciferin intraperitoneally into mice 5 minutes before imaging. The mice were sacrificed at week 6. The *in vivo* liver tumor growth and *ex vivo* lung metastasis were recorded using IVIS 100 Imaging System (Xenogen, Hopkinton, MA). The experiments were performed twice.

### Preparation of protein extracts and western blot analyses

The media of cell culture (both normoxic and hypoxic samples) were removed and rinsed with ice-cold phosphate-buffered saline (PBS). Cellular extracts were collected on ice using RIPA buffer (0.5% sodium deoxycholate, 0.1% sodium dodecyl sulfate, 1% Nonidet P-40, in PBS) supplemented with 1X protease inhibitor cocktail (Roche, Mannheim, Germany). The lysates were incubated for 1 hour on ice and centrifuged for 10 minutes at 14 000 r.p.m. The supernatants were collected and their protein concentrations were determined by the Bradford assay (BioRad, Hercules, CA). Protein lysate was separated by SDS-polyacrylaminde gel electrophoresis (SDS-PAGE) and transferred to polyvinvlidene difluoride membrane (Millipore, Billerica, MA) for western blotting analyses. Primary antibodies against PIM1 (1:1000, Abgent, San Diego, CA), ß-catenin and fibronectin (1: 2500, BD Transduction Laboratories, San Jose, CA), HIF-1α (1:1000, Abcam), vimentin, total AKT, phosp-AKT (Ser473), pyruvate kinase M2 (PKM2) (1:1000, Cell Signaling Technology) ß-actin (1:20000, Sigma-Aldrich), lamin-b (1:10000, Santa Cruz Biotechnology, Santa Cruz, CA) and GAPDH-HRP (1:50000, Abcam) were incubated at 4°C overnight. HRP-conjugated anti-rabbit or anti-mouse IgG were used as secondary antibodies at 1:5000 where appropriate (GE Healthcare Life Sciences, Piscataway, NJ). The signals were visualized using the enhanced chemiluminescence method. For cell fractionation, fresh cells were washed once with ice-cold PBS and centrifuged at 2000 r.p.m. to collect the pellets. The cells (1×10^5^) were lysed with 200 μl protease-inhibitor supplemented hypotonic buffer and incubated on ice for 10 minutes. After adding 2 μl 10% NP40 and vortexed briefly, the lysates were spun at 2500 r.p.m. for 10 minutes. The supernatant were collected as cytoplasmic fraction. The pellets were then washed twice with 500 l 0.9% NaCl solution, followed by RIPA buffer incubation for 1 hour on ice. The cell debris was removed by centrifugation at 14000 r.p.m. for 15 minutes and supernatant collected was the nuclear fraction. GAPDH and lamin-b expression were served as positive control for cytoplasmic and nuclear fractions, respectively. For mouse tumor lysates, the tumors from subcutaneous inoculation were harvested and rinsed with PBS briefly. They were trimmed and snapped frozen using liquid nitrogen. The frozen tumors were disrupted, lysed in RIPA buffer supplemented with protease inhibitor cocktail and phosphatase inhibitor and incubated on ice for four hours. The lysates were vortexed vigorously to facilitate the lyses. Smooth lysates were collected after centrifugation at 14000 r.p.m. for 15 minutes to remove the tissue debris.

### Ubiquitination assay and treatment of DMOG (prolyl hydroxylase inhibitor)

The HCC cells were seeded at 2×10^5^ in 6-well plate 24 hours prior to ubiquitination assay. The cells were treated with proteasome inhibitor MG132 (Sigma-Aldrich) at gradient concentration (0, 5, 10, 20, 30 μM) for 8 hours. DMSO was used as mock control. The protein lysates were collected and upregulation of ß-catenin level marked the success of the inhibition of proteasomal degradation. The experiments were performed twice. Prolyl-4-hydroxylase inhibitor dimethyloxalylglycine (DMOG, Sigma-Aldrich) was applied to inhibit the activity of prolyl hydroxylase (PHD). HCC cells were seeded at 2×10^5^ in 6-well plate 24 hours prior to DMOG treatment at 100 μM for 24 hours. The protein expression of HIF-1α served as positive control. The experiments were performed twice.

### Cell proliferation assay

Proliferation of PIM1 knockdown cells was determined using cell counting method. Either NTC or shPIM1 HCC cells were seeded at 1×10^4^ cells per well into 12-well plate in 1 mL. Total number of cells (for both normoxic and hypoxic conditions) was counted in triplicates each day for five days using Z1 particle counter (Beckman Coulter Inc., Brea, CA). The experiments were performed three times.

### Cell Invasion transwell assay

Invasion Transwell assay was performed as previously described [41]. The cells were incubated in either normoxic (20% O_2_) or hypoxic (1% O_2_) conditions for 16 hours prior to the assay. 1×10^5^ cells in serum free medium were seeded in upper chamber of Matrigel-coated transwell with the lower chamber supplemented with 10% serum medium as chemoattractant. The experiments were harvested after 16 hours. The transwell membranes were fixed with methanol for 1 hour and stained with 1% crystal violet. The membranes were cleaned, air-dried, mounted on slides and documented. The number of cells invaded was counted in 5 random fields. Each experiment was performed in duplicates and for four times.

### Glucose uptake assay

Either stable SMMC-7721 of NTC or shPIM1 group was seeded at 2×10^5^ in 6-well plate and incubated in either normoxic or hypoxic conditions for 24 hours. The media were removed and rinsed twice with PBS to remove the glucose present in the media. The cells were incubated with fluorescent D-glucose analog 2-[N-(7-nitrobenz-2-oxa-1, 3-diazol-4-yl) amino]-2-deoxyglucose (2-NBDG, Invitrogen) at 1:200 diluted in PBS for 15 minutes at 37°C in dark at either 20% or 1% O_2_. The cells were rinsed twice with ice-cold PBS and scrapped out using 1 mL PBS. The 2-NBDG uptake reactions were analyzed using BD FACSCanto II flow cytometer (BD Biosciences) equipped with an argon laser of 488 nm. The results were analyzed by the FlowJo 7.6.3 (Tree Star Inc., Ashland, OR). The experiments were performed twice.

### RNA extraction and quantitative reverse-transcription polymerase chain reaction (qPCR)

Total RNA was extracted using Trizol reagent (Life Technologies) according to manufacturer's protocol. One microgram of RNA was used for cDNA synthesis using GeneAmp RNA PCR Kit (Applied Biosystems, Life Technologies). Gene expression was examined by Power SYBR^®^ Green PCR Master Mix (Applied Biosystems, Life Technologies) using the primers indicated in (Supplementary Table S3) with Applied Biosystems 7900HT Fast Real-Time PCR System. Target gene expressions were normalized with 18s rRNA level.

## SUPPLEMENTARY MATERIAL, FIGURES AND TABLES


