# Supplementary material for: PIM1 regulates glycolysis and promotes tumor progression in hepatocellular carcinoma
Source: Oncotarget. 2015 Mar 12;6(13):10880–92. doi: 10.18632/oncotarget.3534 (PMC4484426; doi:10.18632/oncotarget.3534)
Supplement: Supplementary file 1 [file oncotarget-06-10880-s001.pdf]

PIM1 regulates glycolysis and promotes tumor progression in hepatocellular carcinoma

Supplementary Material

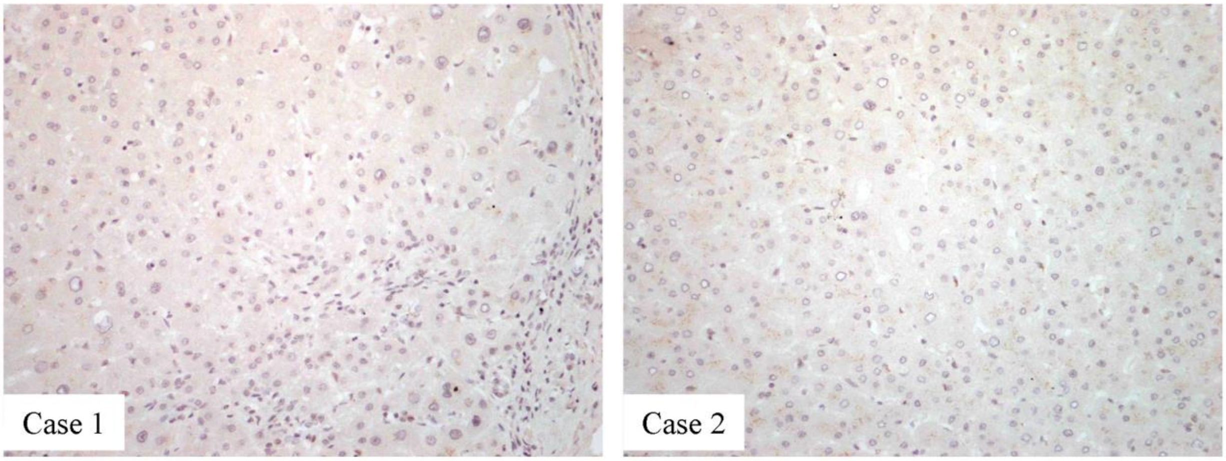

Supplementary Figure 1: PIM1 immunohistochemical staining in liver tissues from liver explant specimens for non-tumoral conditions.

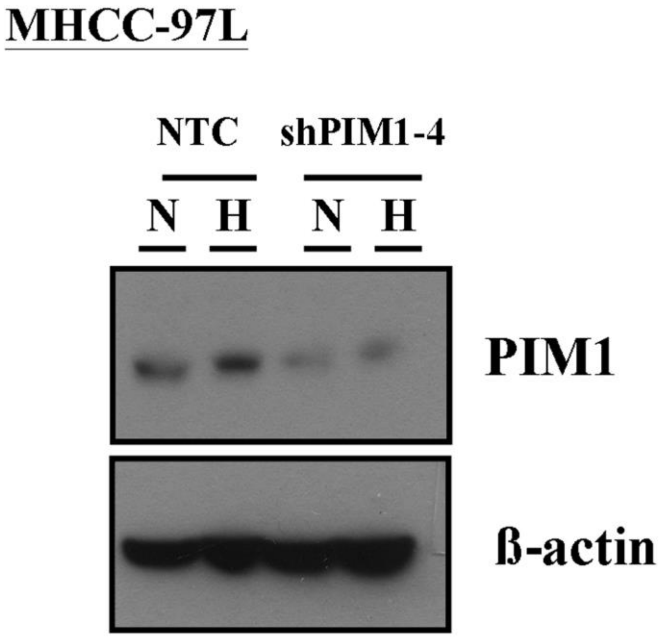

Supplementary Figure 2: Western blot analysis of PIM1 expression in PIM1 stable-knockdown (shPIM1-4) and NTC clones in MHCC-97L cell line.

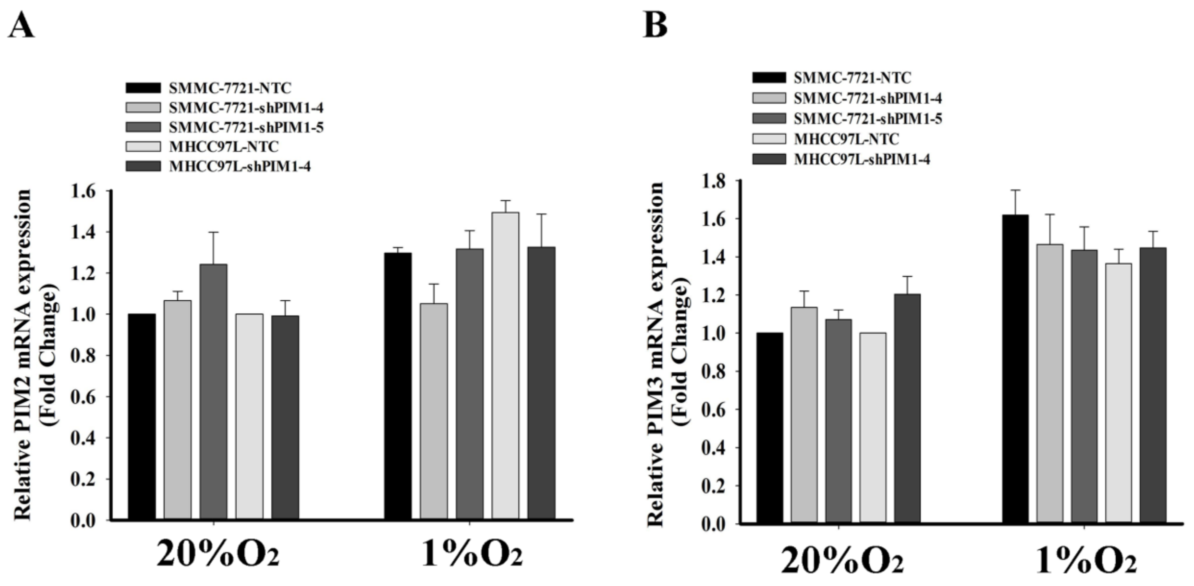

Supplementary Figure 3: Knockdown effect was specific for PIM1 in shPIM1 clones in both SMMC-7721 and MHCC-97L, but not for PIM2 and PIM3. Quantitative PCR analyses for (A) PIM2 and (B) PIM3 in NTC and shPIM1-4 and shPIM1-5 of SMMC-7721 and shPIM1-4 of MHCC-97L.

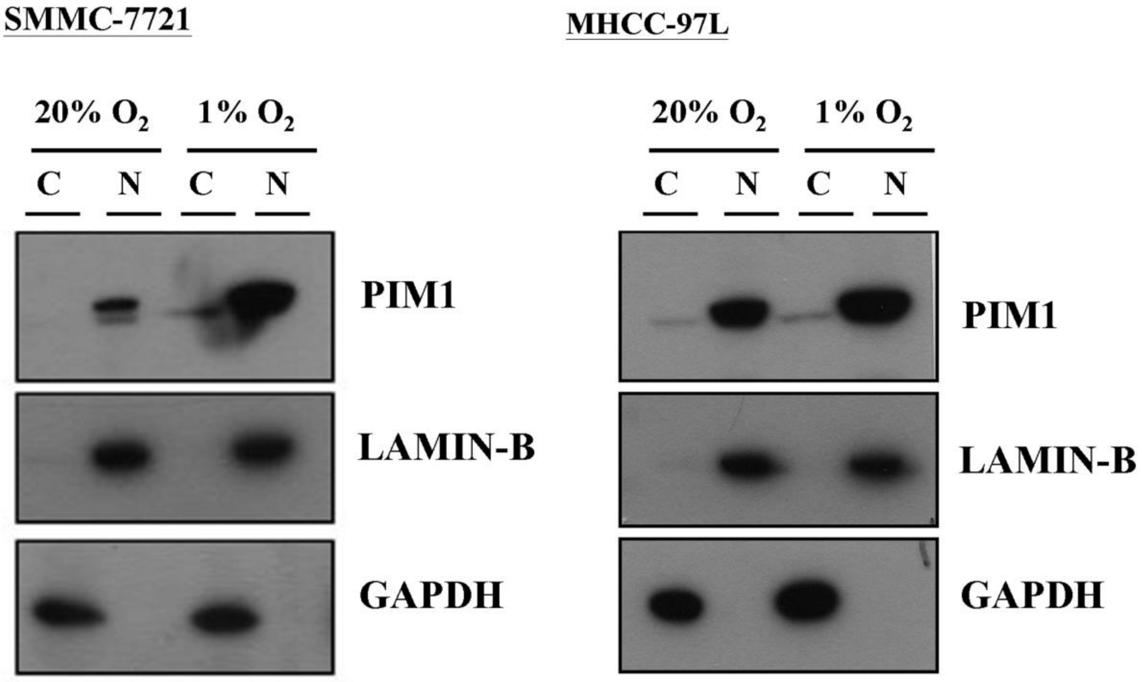

Supplementary Figure 4: Hypoxia enhanced PIM1 expression in the nuclear compartment of HCC cells. Upon hypoxic treatment, the nuclear fraction of PIM1 was markedly increased and predominant in SMMC-7721 and MHCC-97L.

Supplementary Table S1: Clinicopathological features of the primary HCC cohort

Case no.	Number of positive-staining cells%	Nuclear/cytoplasmic staining#	Tumor grading (Edmondson's)	Microvascular invasion	Tumor necrosis	Tumor staging (AJCC)
1	0	-	3	-	-	I
2	0	-	2	+	-	II
3	0	-	2	+	-	II
4	2	N,C	3	+	+	II
5	2	N	2	-	-	I
6	0	-	2	-	-	I
7	0	-	2	-	-	I
8	3	N	3	+	+	II
9	0	-	3	+	-	III
10	0	-	3	-	-	I
11	3	N,C	3	+	+	II
12	0	-	1	-	-	I
13	0	-	3	+	-	II
14	0	-	3	+	-	III
15	0	-	3	+	-	II
16	0	-	2	-	-	I
17	0	-	3	-	-	I
18	0	-	3	-	-	I
19	0	-	2	-	-	I
20	3	N,C	3	+	+	III
21	0	-	2	+	-	II
22	3	N,C	3	+	+	II
23	3	N,C	3	+	+	III
24	3	N,C	3	-	+	III
25	0	-	2	+	-	III
26	2	N,C	3	+	-	III
27	1	N,C	1	-	-	I
28	0	-	2	+	-	III
29	0	-	3	+	-	III
30	0	-	3	+	-	III
31	0	-	1	+	-	II
32	3	N,C	3	+	+	II
33	3	N,C	3	+	+	III
34	0	-	2	+	-	III
35	3	N,C	3	+	-	IV
36	3	N	2	-	-	II
37	0	-	3	+	-	II
38	0	-	2	+	-	III
39	1	N	2	-	+	I
40	0	-	3	-	+	III
41	0	-	2	-	-	I
42	2	N	1	-	+	I
43	0	-	2	+	-	II
44	0	-	2	+	-	II
45	2	N,C	3	+	-	II
46	1	N,C	3	+	-	III
47	1	N	3	-	-	I
48	0	-	3	+	-	III
49	3	N,C	3	+	-	II
50	0	-	2	-	-	I
51	0	-	3	-	-	I
52	3	N,C	3	+	+	II
53	0	-	2	+	-	II
54	0	-	2	-	-	I
55	2	N	3	-	-	I
56	0	-	3	+	+	II

% 1: 1-33%; 2: 34-66%; 3: >67%

N: nuclear; C: cytoplasmic

Supplementary Table S2. Detailed clinicopathological features of the two cohorts with paired primary and metastatic HCC samples

Case no.	Primary tumor					Metastatic tumor			
	Number of positive-staining cells%	Nuclear/cytoplasmic staining#	Tumor grading (Edmondson's)	Microvascular invasion	Tumor staging (AJCC)^	Number of positive-staining cells	Nuclear/cytoplasmic staining#	Tumor grading (Edmondson's)	Time interval@ (months)
1	3	N	3	+	II	3	N,C	3	117
2	3	N,C	3	+	II	3	N	3	23
3	0	-	3	+	II	3	N	3	8
4	0	-	2	-	II	0	-	2	4
5	0	-	2	-	I	3	N	2	21
6	2	N	3	+	IV	2	N	3	3
7	1	N,C	3	-	III	3	N,C	3	13
8	1	N	3	-	II	3	N	3	28
9	0	-	3	+	II	3	N	3	13
10	0	-	2	-	II	3	N	3	5
11	1	N	3	+	IV	2	N	3	0
12	2	N,C	3	+	IV	3	N,C	3	0
13	0	-	3	-	I	2	N	3	28
14	3	N,C	3	-	I	3	N,C	3	43
15	0	-	2	+	II	3	N,C	3	17
16	0	-	2	-	I	3	N,C	3	34
17	1	N	2	-	I	3	N,C	2	8
18	1	N,C	3	-	I	2	N,C	3	21
19	2	N	2	+	IV	3	N	2	0
20	0	-	3	-	I	3	N,C	3	97
21	0	-	3	+	IV	0	-	3	0
22	0	-	3	+	IV	2	N	3	0
23	0	-	2	+	IV	0	-	2	0
24	0	-	3	+	II	3	N	3	48
25	0	-	2	+	IV	0	-	3	0
26	0	-	3	-	III	3	N	3	9
27	1	N	3	+	II	0	-	3	26
28	0	-	3	+	II	0	-	3	62
29	0	-	3	+	III	3	N	3	8
30	0	-	2	+	II	0	-	3	17
31	0	-	2	+	II	0	-	2	16
32	0	-	3	-	III	0	-	3	28
33	0	-	3	-	IV	1	N	3	0
34	0	-	3	+	III	0	-	3	22
35	0	-	3	-	IV	0	-	3	0
36	3	N	3	+	II	3	N,C	3	8
37	0	-	3	+	IV	2	N	3	0
38	1	N,C	3	+	IV	1	N,C	3	0
39	0	-	2	+	II	0	-	2	68
40	0	-	3	+	III	0	-	3	7
41	0	-	2	-	I	2	N,C	2	14
42	1	N	3	+	III	3	N	3	27
43	0	-	3	+	II	3	N	3	74

% 1: 1-33%; 2: 34-66%; 3: >67%
\# N: nuclear; C: cytoplasmic
^ Tumor staging at diagnosis of primary tumor
@ time elapsed between resection of primary tumor and metastasis

Supplementary Table S3: Primer sequences for qPCR

	**<u>Genes</u>**		
**1**	**PIM1**	Forward	5' CTCGCCGCGATTGAGGTCGATAA 3'
		Reverse	5' CAACTTGCCGGTGGCCATCAAACA 3'
**2**	**PIM2**	Forward	5' CCATTCCCGTGGAGTTGT 3'
		Reverse	5' GAAGCAGGGCACCAGAAC 3'
**3**	**PIM3**	Forward	5' CGGAGGAGGGTCTCTCCAGAGTG 3'
		Reverse	5' ACCCTGCGCCGGCGGAAAG 3'
**4**	**PKM2**	Forward	5' ATCGTCCTCACCAAGTCTGG 3'
		Reverse	5' GAAGATGCCACGGTACAGGT 3'
**5**	**GLUT-1**	Forward	5' CGGGCCAAGAGTGTGCTAAA 3'
		Reverse	5' TGACGATACCGGAGCCAATG 3'
**6**	**18s rRNA**	Forward	5' GTAACCCGTTGAACCCCATT 3'
		Reverse	5' CCATCCAATCGGTAGTAGCG 3'
